# Association between serum lactate dehydrogenase level and renal outcome in patients with advanced chronic kidney disease without diabetes mellitus

**DOI:** 10.1007/s10157-026-02855-4

**Published:** 2026-04-15

**Authors:** Yoshihiro Nakamura, Shimon Kurasawa, Takahiro Imaizumi, Terumasa Hayashi, Ichiei Narita, Kenichiro Tanabe, Satoshi Morita, Yoshiharu Tsubakihara, Enyu Imai, Tadao Akizawa, Shoichi Maruyama

**Affiliations:** 1 Department of Nephrology, Nagoya University Graduate School of Medicine, 65 Tsurumai-cho, Showa-ku, Nagoya, 466-8550 Aichi Japan; 2 Department of Advanced Medicine, Nagoya University Hospital, Nagoya, Aichi Japan; 3 Department of Kidney Disease and Hypertension, Osaka General Medical Center, Osaka, Japan; 4 Division of Clinical Nephrology and Rheumatology, Niigata University, Niigata, Japan; 5 Division of Health Data Science, Translational Research Center for Medical Innovation, Kobe, Japan; 6 Pathophysiology and Bioregulation, St. Marianna University Graduate School of Medicine, Kanagawa, Japan; 7 Biomedical Statistics and Bioinformatics, Kyoto University, Kyoto, Japan; 8 Graduate School of Health Care Sciences, Jikei Institute, Osaka, Japan; 9 Nakayamadera Imai Clinic, Takarazuka, Japan; 10 Division of Nephrology, Showa University School of Medicine, Tokyo, Japan

**Keywords:** Advanced chronic kidney disease, Cardiovascular events, Lactate dehydrogenase, Renal outcome

## Abstract

**Background:**

Higher serum lactate dehydrogenase (LDH) levels are associated with poor prognosis including renal outcome, all-cause and cardiovascular mortality in patients with diabetic kidney disease. However, this association remains unclear in patients with chronic kidney disease (CKD) without diabetes mellitus (DM).

**Methods:**

Using data from the PREDICT trial, which enrolled patients aged 20–85 years with an estimated glomerular filtration rate (eGFR) of 8–20 mL/min/1.73 m^2^ and hemoglobin <10 g/dL due to renal anemia without DM, we investigated the association between serum LDH levels and study outcomes.

**Results:**

In total, 444 patients (median age, 73 years; 61% men; mean eGFR, 13.5 mL/min/1.73 m^2^) were divided into four quartiles according to serum LDH level: Q1, 78–169 U/L; Q2, 170–193 U/L; Q3, 194–224 U/L; Q4, 225–503 U/L. The median observation period for renal composite events and the composite of cardiovascular events or death were 562 and 637 days, respectively. In total, 206 (46%) patients experienced renal composite outcomes, and 45 (10%) patients experienced a composite of cardiovascular events or death. The multivariable analysis showed that the Q4 group had a higher risk of renal composite outcomes (adjusted hazard ratio [aHR], 2.07; 95% confidence interval [CI], 1.39–3.08) and the composite of cardiovascular events or death (aHR, 2.77; 95%CI, 1.21–6.32) compared with the Q1 group.

**Conclusions:**

Higher LDH levels are associated with higher risk of renal complications and composite of cardiovascular events or death in patients with advanced CKD without DM.

**Supplementary Information:**

The online version contains supplementary material available at 10.1007/s10157-026-02855-4.

## Introduction

Worldwide, a large number of patients with chronic kidney disease (CKD) require dialysis, and the high risk of cardiovascular (CV) events and mortality in patients with CKD makes a significant global health issue [1, 2]. Thus, various biomarkers have been studied to predict the onset and progression of CKD [3, 4]; however, many of these biomarkers are not easily measured in daily practice.

As lactate dehydrogenase (LDH) is an enzyme distributed intracellularly in several tissues, and an elevated serum LDH level reflects cellular damage [5, 6]. Serum LDH levels are elevated in various diseases, including malignancy, liver disease, pneumocystis jiroveci pneumonia, and COVID-19 [7–10]; moreover, higher serum LDH level is a risk factor for mortality in these patients [8, 11–13]. Regarding the pathophysiology of elevated serum LDH levels, tumor cells release intracellular enzymes, including LDH, through the damaged cell membrane [5]. Another explanation is that LDH reflect inflammation of the tissue in patients with cancer or some infectious diseases [5, 10].

Higher serum LDH level is associated with poor renal outcomes and all-cause and CV mortality in patients with diabetic kidney disease (DKD) [14]; thus, serum LDH may be a useful prognostic marker in patients with DKD. However, whether higher serum LDH level is associated with renal and CV outcomes in patients with CKD without diabetes mellitus (DM) remains unclear; thus, this aspect was examined using data from the prevention of end-stage kidney disease by darbepoetin alfa in CKD patients with non-diabetic kidney disease (PREDICT) trial.

## Methods

### Study population

This study was conducted using data from the PREDICT trial. The study design and details have been previously reported [15, 16]. Briefly, this was a multicenter, randomized, open-label, parallel-group study conducted in patients with advanced CKD and anemia without DM. Between December 2011 and June 2014, 491 patients aged 20–85 years with an estimated glomerular filtration rate (eGFR) of 8–20 mL/min/1.73 m^2^ and hemoglobin <10 g/dL due to renal anemia were enrolled from 74 institutions in Japan. Patients were excluded if they had DM, uncontrolled hypertension (systolic blood pressure ≥180 mmHg or diastolic blood pressure ≥110 mmHg), advanced congestive heart failure (New York Heart Association class III or IV), malignancy, hematological disorders, hemorrhagic lesions, malnutrition, ongoing bleeding from gastrointestinal ulcers, acute infections, collagen vascular diseases, or severe allergies. Patients were randomly assigned in a 1:1 ratio to either the low-hemoglobin (9–11 g/dL) or high-hemoglobin (11–13 g/dL) treatment group to investigate the impact of anemia management on subsequent renal outcomes. In this secondary analysis to investigate the relationship between serum LDH level and study outcomes, patients in whom serum LDH level information was unavailable were excluded. The PREDICT trial was designed, conducted, and monitored by the PREDICT Executive Committee, and the study was registered at Clinicaltrials.gov (registration number NCT01581073).

### Baseline variables

History of CV disease (CVD) was defined as the presence of any of the following conditions: acute myocardial infarction, heart failure, stroke, or peripheral artery disease. The eGFR was calculated using the Japanese Society of Nephrology’s equation as follows:

$${\mathrm{eGFR}} = 194 \times {\mathrm{serum}}\,{\kern 1pt} {\mathrm{creatinine}}^{ - 1.094} \times {\mathrm{age}}^{ - 0.287} ( \times 0.739\,{\mathrm{for}}\,{\mathrm{women}})$$

The etiology of chronic kidney disease is determined by the nephrologist’s judgment.

### Exposure

Patients were divided into four quartiles based on their serum LDH levels (Q1, Q2, Q3, and Q4). The exposure of interest was a higher serum LDH level (Q2–Q4) compared with the lowest serum LDH level (Q1).

### Outcomes

The primary outcome was the renal composite outcome (initiation of maintenance dialysis treatment; kidney transplantation; eGFR reduction to ≤6 mL/min per 1.73 m^2^; or ≥50% reduction in eGFR from baseline). Secondary outcome was defined as a composite of CV events and all-cause death. CV events were defined as myocardial infarction, stroke, hospitalization for heart failure, hospitalization for angina, or amputation of the lower extremities. Patients were followed up from the time of enrollment until death, initiation of renal replacement therapy, failure to follow-up, inability to continue darbepoetin-alfa according to the protocol, or the end of the follow-up period (2 years from the baseline date).

### Statistical analyses

The cohort was divided into four groups according to serum LDH levels. For baseline data, continuous variables were summarized as means ± standard deviation (SD) or medians (interquartile range [IQR]), and categorical variables were summarized as numbers (percentages). The correlations between serum LDH level and clinical parameters were analyzed using correlation analysis. For normally distributed numerical variables, Pearson correlation was employed, while Spearman correlation was used for the other variables. Kaplan–Meier survival curves for the renal composite outcome and the composite of CV events or death were plotted across the four groups. Differences in the survival estimates among the four groups were evaluated using the log-rank test. Cox proportional hazards models were developed to calculate the hazard ratio (HR) with a 95% confidence interval (CI), facilitating the comparison of outcomes across the four groups using the Q1 group as a reference. Three models were explored: Model 1 was unadjusted, Model 2 was adjusted for age and sex, and Model 3 additionally accounted for history of CVD, eGFR, serum hemoglobin level, serum C-reactive protein (CRP) level, urine protein creatinine ratio (<0.15, 0.15–0.49, and ≥0.5 g/gCr), and the treatment group (low- and high-hemoglobin). Serum CRP levels were log-transformed when included in the models. The association between serum LDH levels as continuous variables and HRs for the renal composite outcome and the composite of CV events or death were visualized using restricted cubic splines with three knots. The references were set at 200 U/L, which is the mean of this study. To handle the missing data, we performed an analysis using multiple imputations with chained equations in a multivariable model. Twenty imputations were made, and the estimates of the analysis for each dataset were combined using Rubin’s rule [17]. All the variables listed in Table 1 were included in the imputation model [18].

**Table 1:** Baseline characteristics by the study group

	Total	Q1 (LDH 78–169 U/L)	Q2 (LDH 170–193 U/L)	Q3 (LDH 194–224 U/L)	Q4 (LDH 225–503 U/L)	Missing (*n*)
	*n* = 444	*n* = 112	*n* = 111	*n* = 112	*n* = 109	
Age^a^, years	73 (65–79)	70 (58–78)	74 (66–78)	72 (65–78)	75 (68–81)	0
Male sex	270 (61%)	77 (69%)	65 (59%)	65 (58%)	63 (58%)	0
Body mass index, kg/m^2^	22.4 ± 3.2	22.4 ± 3.6	22.3 ± 2.8	22.4 ± 3.4	22.3 ± 3.2	25
Current or former smoking	69 (17%)	13 (12%)	18 (17%)	18 (18%)	20 (20%)	34
Etiology of chronic kidney disease						0
Chronic glomerulonephritis	135 (30%)	34 (30%)	34 (31%)	39 (35%)	28 (26%)	
Nephrosclerosis	230 (52%)	53 (47%)	59 (53%)	54 (48%)	64 (59%)	
Others	79 (18%)	25 (22%)	18 (16%)	19 (17%)	17 (16%)	
History of cardiovascular disease	82 (18%)	21 (19%)	20 (18%)	17 (15%)	24 (22%)	0
Systolic blood pressure, mmHg	133 ± 16	130 ± 17	132 ± 14	132 ± 17	136 ± 17	0
Diastolic blood pressure, mmHg	72 ± 11	73 ± 12	71 ± 11	73 ± 12	71 ± 11	0
Pulse pressure^a^, mmHg	61 ± 15	57 ± 15	61 ± 14	59 ± 13	65 ± 16	0
Hemoglobin, g/dL	9.3 ± 0.6	9.3 ± 0.6	9.4 ± 0.6	9.3 ± 0.6	9.2 ± 0.6	0
Ferritin, ng/mL	150 (100–238)	136 (91–232)	143 (97–203)	162 (105–261)	167 (97–266)	6
Transferrin saturation, %	33 ± 11	33 ± 12	33 ± 11	34 ± 12	31 ± 10	11
Urea nitrogen^a^, mg/dL	50.4 ± 15.2	46.9 ± 13.7	49.9 ± 15.3	51.6 ± 15.6	53.5 ± 15.6	0
Creatinine^a^, mg/dL	3.6 ± 1.0	3.8 ± 1.0	3.5 ± 0.9	3.5 ± 1.1	3.5 ± 1.0	0
eGFR, mL/min/1.73m^2^	13.5 ± 3.3	13.1 ± 3.2	13.6 ± 3.2	13.8 ± 3.4	13.6 ± 3.5	0
Potassium, mEq/L	4.8 ± 0.6	4.8 ± 0.6	4.9 ± 0.7	4.8 ± 0.6	4.8 ± 0.7	1
Calcium^a^, mg/dL	8.7 ± 0.5	8.8 ± 0.5	8.7 ± 0.5	8.8 ± 0.5	8.6 ± 0.6	0
Phosphorus, mg/dL	4.0 ± 0.8	3.9 ± 0.8	3.9 ± 0.7	4.0 ± 0.8	4.1 ± 0.8	8
Albumin, g/dL	3.8 ± 0.4	3.8 ± 0.4	3.8 ± 0.4	3.8 ± 0.4	3.8 ± 0.4	4
C-reactive protein, mg/dL	0.09 (0.03–0.21)	0.10 (0.04–0.21)	0.10 (0.03–0.25)	0.08 (0.02–0.21)	0.10 (0.03–0.20)	40
Aspartate aminotransferase^a^, U/L	19 ± 9	15 ± 6	19 ± 8	20 ± 9	23 ± 11	3
Alanine aminotransferase^a^, U/L	14 ± 8	11 ± 6	13 ± 7	14 ± 8	17 ± 11	2
Low-density lipoprotein cholesterol^a^, mg/dL	97 ± 28	92 ± 24	94 ± 27	98 ± 27	103 ± 32	37
High-density lipoprotein cholesterol^a^, mg/dL	51 ± 17	45 ± 13	52 ± 21	53 ± 16	53 ± 17	45
Proteinuria^a^, g/gCr	1.2 (0.5–2.3)	0.8 (0.4–1.8)	1.1 (0.6–2.1)	1.2 (0.5–2.3)	1.7 (0.5–3.3)	0
Proteinuria group, g/gCr						0
<0.15	31 (7.0%)	9 (8.0%)	7 (6.3%)	7 (6.3%)	8 (7.3%)	
0.15–0.49	85 (19%)	31 (28%)	17 (15%)	19 (17%)	18 (17%)	
≥0.5	328 (74%)	72 (64%)	87 (78%)	86 (77%)	83 (76%)	
Antihypertensive drugs	407 (92%)	98 (88%)	102 (92%)	107 (96%)	100 (92%)	0
ACEIs or ARBs	305 (69%)	72 (64%)	81 (73%)	82 (73%)	70 (64%)	0
Lipid-lowering drugs	165 (37%)	35 (31%)	43 (39%)	47 (42%)	40 (37%)	0
High-hemoglobin treatment group	221 (50%)	53 (47%)	51 (46%)	62 (55%)	55 (50%)	0

Values are expressed as mean ± SD or median (interquartile range) for continuous measures, and number (percent) for categorical measures

^a^ *p* < 0.05 for Student’s *t*-test, Wilcoxon rank sum test, or chi-square test

*ACEIs* Angiotensin converting enzyme inhibitors, *ARBs* Angiotensin II receptor blockers, *eGFR* Estimated glomerular filtration rate, *LDH* Lactate dehydrogenase

Subgroup analyses according to treatment group (low- and high-hemoglobin), age (<70 and ≥70 years), sex, and etiology of chronic kidney disease (chronic glomerulonephritis, nephrosclerosis, and others) for renal composite outcomes were performed. Interaction was calculated using the Wald test.

In all analyses, a two-sided *p*-value of <0.05 was considered statistically significant. All the statistical analyses were performed using Stata version 17 (StataCorp, College Station, TX, USA).

## Results

### Patient characteristics

Of these 491 patients, 444 were included in the survival analysis (Suppl. Fig. 1). The participants’ characteristics are listed in Table 1. Overall, the median [IQR] age was 73 [65–79] years, 61% were men, the mean eGFR was 13.5 mL/min/1.73 m^2^, and >90% of patients were using antihypertensive drugs. The Q1, Q2, Q3, and Q4 groups had serum LDH levels of 78–169, 170–193, 194–224, and 225–503 U/L, respectively. The Q4 group had the highest pulse pressure and proteinuria. In addition, serum aspartate aminotransferase and alanine aminotransferase levels were highest in the Q4 group, although both were within the normal range. The high-hemoglobin treatment group did not differ among the four groups. Missing data were found for <11% of each variable.

### Correlations of serum LDH level with clinical parameters

Serum LDH levels were weakly positively correlated with age (*r* = 0.16; *p* < 0.001) and proteinuria (*r* = 0.17; *p* < 0.001), but not with serum hemoglobin (*r* = −0.03; *p* = 0.46), eGFR (*r* = 0.10; *p* = 0.099), or log-transformed CRP (*r* = −0.07; *p* = 0.26) (Suppl. Fig. 2).

### Associations between higher serum LDH level and study outcomes

**Fig. 1:**
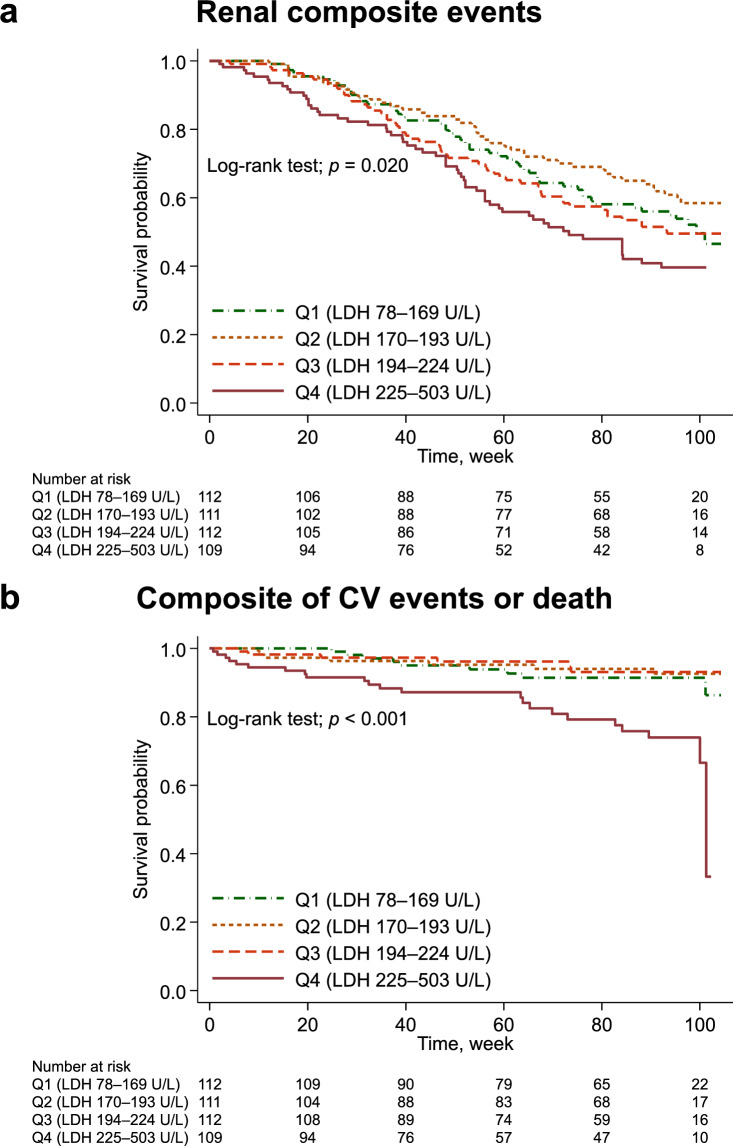
Time-to-event curves for study outcomes. The Q4 group had a higher risk of both (**a**) renal composite events and (**b**) the composite of CV events or death. *CV* Cardiovascular, *LDH* Lactate dehydrogenase

The median [IQR] observation period for renal composite events was 562 [288–680] days, and 206 (46%) patients experienced renal composite events. During follow-up, there were 45 (10%) censored cases, comprising 33 patients who were lost to follow-up and 12 patients who were unable to continue darbepoetin alfa treatment according to the protocol. Kaplan–Meier curves showed a higher number of renal composite events in the Q4 group compared with the other groups (*p* = 0.020; Fig. 1a). The multivariable analysis showed that the Q4 group (aHR, 2.07; 95%CI, 1.39–3.08) had higher risk of renal composite events than the Q1 group, whereas the risk in the Q2 (aHR, 0.97; 95%CI, 0.63–1.49) and Q3 (adjusted HR [aHR], 1.40; 95%CI, 0.94–2.08) groups were not statistically different from that in the Q1 group (Table 2).

**Table 2:** Outcomes according to the study group

	Q1 (LDH 78–169 U/L)	Q2 (LDH 170–193 U/L)	Q3 (LDH 194–224 U/L)	Q4 (LDH 225–503 U/L)
	*n* = 112	*n* = 111	*n* = 112	*n* = 109
Renal composite events^a^, *n* (%)	51 (46%)	42 (38%)	54 (48%)	59 (54%)
Maintenance dialysis treatment, *n* (%)	42 (38%)	35 (32%)	49 (44%)	51 (47%)
Kidney transplantation, *n* (%)	4 (3.6%)	1 (0.9%)	0 (0%)	0 (0%)
50% reduction in eGFR, *n* (%)	21 (19%)	14 (13%)	23 (21%)	20 (18%)
eGFR of ≤6 mL/min per 1.73 m^2^, *n* (%)	25 (22%)	16 (14%)	19 (17%)	22 (20%)
Rate,/100 person-years (95%CI)	32.8 (24.9–43.1)	26.5 (19.5–35.8)	35.7 (27.4–46.6)	46.4 (36.0–59.9)
Unadjusted HR (95%CI)	1.00 (reference)	0.80 (0.53–1.20)	1.10 (0.75–1.61)	1.47 (1.01–2.14)
Age- and sex-adjusted HR (95%CI)	1.00 (reference)	0.88 (0.58–1.32)	1.27 (0.86–1.87)	1.88 (1.29–2.76)
Multivariable-adjusted HR (95%CI)	1.00 (reference)	0.97 (0.63–1.49)	1.40 (0.94–2.08)	2.07 (1.39–3.08)
Cardiovascular events or death^b^, *n* (%)	9 (8.0%)	7 (6.3%)	6 (5.4%)	23 (21%)
Cardiovascular events, *n* (%)	7 (6.2%)	6 (5.4%)	4 (3.6%)	15 (14%)
Death, *n* (%)	6 (5.4%)	3 (2.7%)	2 (1.8%)	12 (11%)
Rate,/100 person-years (95%CI)	5.5 (2.9–10.7)	4.3 (2.1–9.1)	3.8 (1.7–8.5)	17.6 (11.7–26.5)
Unadjusted HR (95%CI)	1.00 (reference)	0.80 (0.30–2.14)	0.69 (0.25–1.94)	3.33 (1.53–7.22)
Age- and sex-adjusted HR (95%CI)	1.00 (reference)	0.67 (0.25–1.81)	0.62 (0.22–1.74)	2.57 (1.17–5.63)
Multivariable-adjusted HR (95%CI)	1.00 (reference)	0.58 (0.21–1.63)	0.65 (0.23–1.87)	2.77 (1.21–6.32)

The multivariable-adjusted model was adjusted for age, sex, history of cardiovascular disease, estimated glomerular filtration rate, hemoglobin, C-reactive protein, proteinuria, and high-hemoglobin treatment group

^a^Renal composite events reflects the first occurrence of any of its components

^b^Cardiovascular events or death reflects the first occurrence of these components

*CI* Confidence interval, *eGFR* Estimated glomerular filtration rate, *HR* Hazard ratio, *LDH* Lactate dehydrogenase

The median [IQR] observation period for the composite of CV events or death was 637 [325–682] days, and 45 patients (10%) experienced a composite of CV events or death. Kaplan–Meier curves showed a higher incidence in the composite of CV events or death in the Q4 group (*p* < 0.001; Fig. 1b). The multivariable analysis showed that the Q4 group (aHR, 2.77; 95%CI, 1.21–6.32) was associated with a higher risk of composite CV events or death compared with the Q1 group, whereas the risk was not statistically different in the Q2 (aHR, 0.58; 95%CI, 0.21–1.63) and Q3 (aHR, 0.65; 95%CI, 0.23–1.87) groups compared with that in the Q1 group (Table 2).

The spline analyses revealed that the risk of renal composite outcome and the composite of CV events or death increased as the serum LDH levels increased (Suppl. Fig. 3).

### Subgroup analyses

**Fig. 2:**
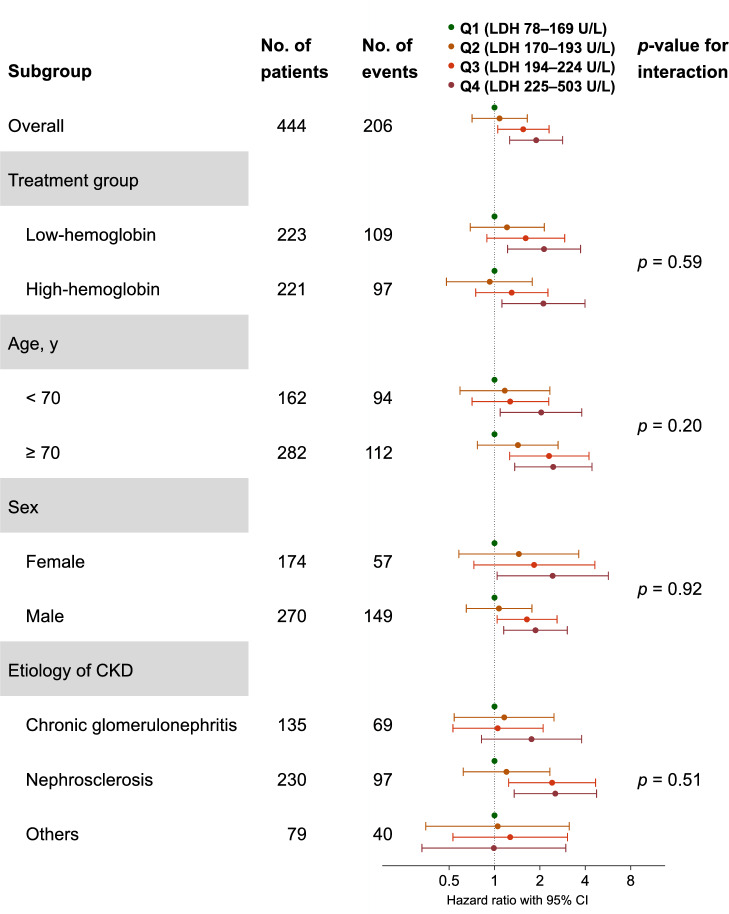
Subgroup analyses of renal composite outcomes. Plots with capped spikes show multivariable-adjusted hazard ratios with 95%CIs for renal composite outcomes of groups with higher LDH levels (Q2–Q4) compared with that in the lowest LDH group (Q1) as reference. Low-hemoglobin treatment group was assigned to a hemoglobin level of 9–11 g/dL, and the high-hemoglobin treatment group was assigned to a hemoglobin level of 11–13 g/dL. Patients with nephrosclerosis and higher serum LDH levels (Q3 and Q4) tended to have higher risks of renal composite events than those in the other groups, although the interaction was not significant. *CI* Confidence interval, *CKD* Chronic kidney disease, *LDH* Lactate dehydrogenase

The association among the four groups and renal composite events was mostly consistent across the subgroups. Although the interaction was not significant, patients with nephrosclerosis and higher serum LDH levels (Q3 and Q4) tended to have higher risks of renal composite events than those in the other groups (Fig. 2).

## Discussion

Using data from the PREDICT trial, we examined the association between serum LDH level and both renal composite outcomes and the composite of CV events or death. A higher serum LDH level was a risk factor for poor renal composite outcomes in patients with advanced CKD without DM. Furthermore, higher serum LDH levels may also be a risk factor for CV events or death in these patients.

In patients with DM, higher serum LDH levels were associated with the presence of DKD [19]. Furthermore, higher serum LDH levels were associated with poor renal outcomes in patients with DKD [14]; however, to our knowledge, there are no reports examining renal outcomes in patients with CKD without DM. Our results suggest that higher serum LDH levels are a risk factor for poor renal composite outcomes in patients with advanced CKD without DM (mean eGFR: 13.5 mL/min/1.73 m^2^). In the past report about DKD [14], there is a significant difference in eGFR between the two groups (median eGFR: 73.2 mL/min/1.73m^2^ for the lower LDH group and median eGFR: 55.2 ± 3.2 mL/min/1.73m^2^ for the higher LDH group). The strength of this study is that the eGFR level was comparable across the four groups. In our study, serum LDH levels >225 U/L were associated with poor renal prognosis in patients with CKD without DM. While the reference range for serum LDH varies by institution, a value of 225 U/L approximately corresponds to a level exceeding the upper limit of normal. Thus, values above the upper limit of normal are considered to be associated with a high risk. However, spline analysis indicated a linear relationship between serum LDH levels and renal composite outcomes; thus, future studies with larger sample sizes are warranted. Serum LDH can be easily measured in daily practice; therefore, it is useful for identifying high-risk patients with progressive kidney deterioration in advanced CKD without DM.

Previously, a higher serum LDH level was identified as a risk factor for CV and all-cause mortality in patients with DKD and in those initiating hemodialysis [14, 20]. In our study, among patients with advanced CKD without DM, the highest serum LDH level (Q4) group had a higher risk of composite CV events or death than the Q1 group. This suggests that a higher serum LDH level is a risk factor for CV events or death. In contrast, the HRs of the Q2 and Q3 groups were <1; however, no statistically significant difference was observed. In patients with advanced CKD without DM, active screening for CVD may be beneficial in cases of higher serum LDH levels; however, further studies are needed to draw conclusions.

A possible explanation for the present result—that a higher serum LDH level (Q4) is associated with renal and CV outcomes—is that higher serum LDH levels may reflect systemic vascular endothelial cell damage. Hypertension causes vascular endothelial dysfunction [21]. Furthermore, the frequency of vascular endothelial dysfunction increases as serum LDH levels increase in patients with hypertension [22]. Serum LDH is considered a sensitive indicator of cellular damage [6]; thus, in our study, which included >90% of patients on antihypertensive drugs, higher serum LDH levels may reflect damage to endothelial cells [22, 23]. The pulse pressure was highest in the LDH (Q4) group, which also suggests systemic endothelial cell damage [24].

In the subgroup analysis, the relationship between higher serum LDH level and renal composite outcomes was stronger in patients with nephrosclerosis than that in those with other etiologies of CKD, although the interaction was not significant. Thus, it is possible that longstanding hypertension might be particularly associated with endothelial cell damage in our study, although it is known that other causes of endothelial cell damage include smoking and lipid abnormalities [25, 26]. Furthermore, because serum LDH levels correlate with proteinuria, we hypothesized that this result is possibly due to glomerular endothelial cell damage. Serum LDH levels were not correlated with log-transformed CRP; however, the possibility remains that higher serum LDH levels may reflect inflammation. Future research that identifies factors contributing to higher serum LDH levels in advanced CKD without DM may deepen our understanding of the mechanisms underlying the progression of renal impairment and may contribute to the development of treatments.

This study has some limitations. First, the sample size was relatively small (*n* = 444). In total, 206 patients had a renal composite outcome; however, there were only 45 patients with composite CV events or death, resulting in relatively wide CIs. Second, as we only included patients with an eGFR of <20 mL/min/1.73 m^2^, we cannot conclude whether measuring serum LDH levels is useful in patients with eGFR of ≥20 mL/min/1.73 m^2^. Third, because the dose of erythropoiesis-stimulating (ESA) agent needed to maintain a target hemoglobin level differs substantially among individuals, the potential impact of high-dose ESA use or ESA hyporesponsiveness on the outcomes cannot be excluded. Thus, the subgroup analysis was conducted for renal composite outcomes according to treatment group (low- and high-hemoglobin), and the result was comparable in both treatment groups. Forth, in this study, the etiology of CKD is determined by the nephrologist’s judgment. This may have affect the result of the subgroup analysis. However, we believe that its impact on the main analysis was small. Further studies on the early stages of CKD in patients without DM are needed.

In conclusion, higher serum LDH levels are associated with higher risks of renal complications and composite CV events or death in patients with advanced CKD without DM. Further studies are needed to gain a comprehensive understanding of the pathophysiology of this result.

## Supplementary Information

Below is the link to the electronic supplementary material.

Supplementary file1 (DOCX 485 KB)
