# Supplementary material for: Association between serum lactate dehydrogenase level and renal outcome in patients with advanced chronic kidney disease without diabetes mellitus
Source: Clin Exp Nephrol. 2026 Apr 15;30(6):905–13. doi: 10.1007/s10157-026-02855-4 (PMC13242488; doi:10.1007/s10157-026-02855-4)
Supplement: Supplementary file 1 — Supplementary file1 (DOCX 485 KB) [file 10157_2026_2855_MOESM1_ESM.docx]

**Supplementary Material for:**

**Association between serum lactate dehydrogenase level and renal outcome in patients with advanced chronic kidney disease without diabetes mellitus**

**Table of Contents:**

[Figure S1. Flow diagram illustrating patient enrollment for the present study. 2](#_Toc162261843)

[Figure S2. Correlations of serum LDH levels with each clinical parameters 3](#_Toc162261844)

Figure S3. Restricted cubic spline analysis to compare the association of serum LDH levels with the study outcomes 4

**Figure S1. Flow diagram illustrating patient enrollment for the present study**


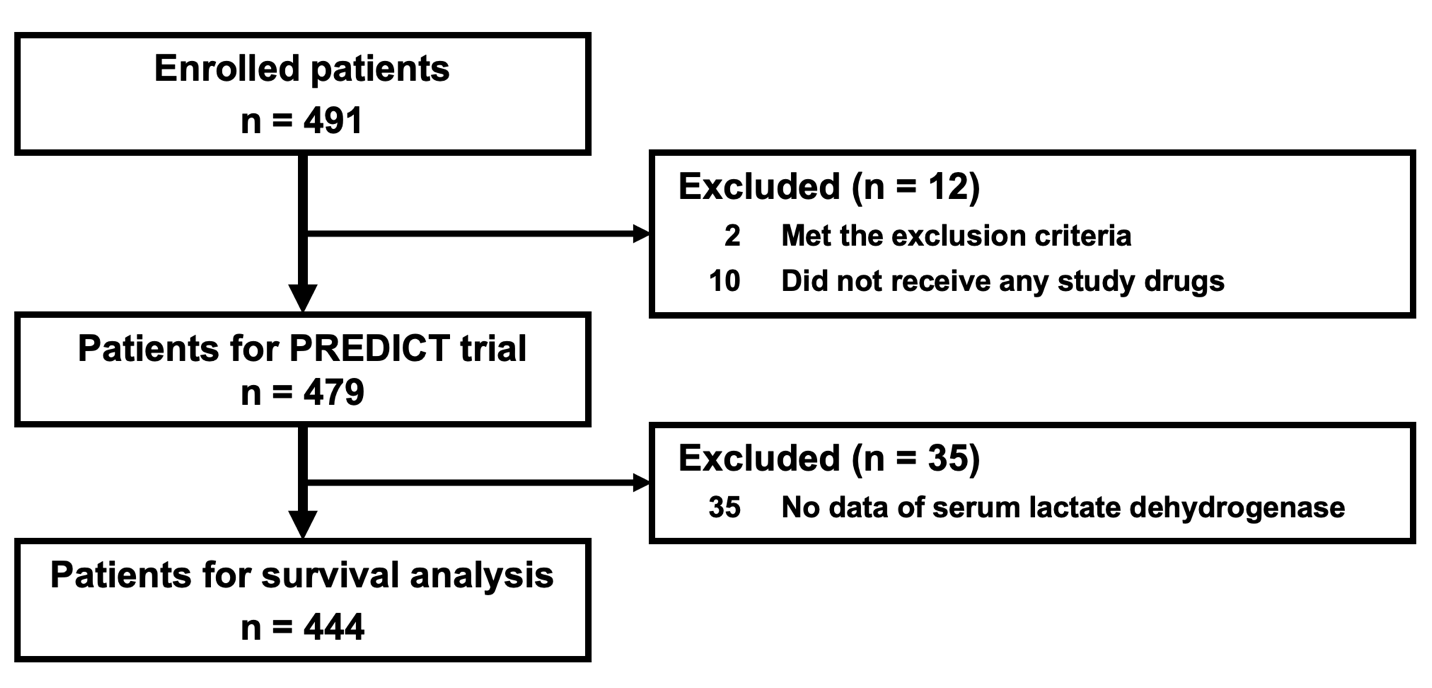


A total of 444 patients were included in the survival analysis.

**Figure S2. Correlations of serum LDH levels with each clinical parameters**


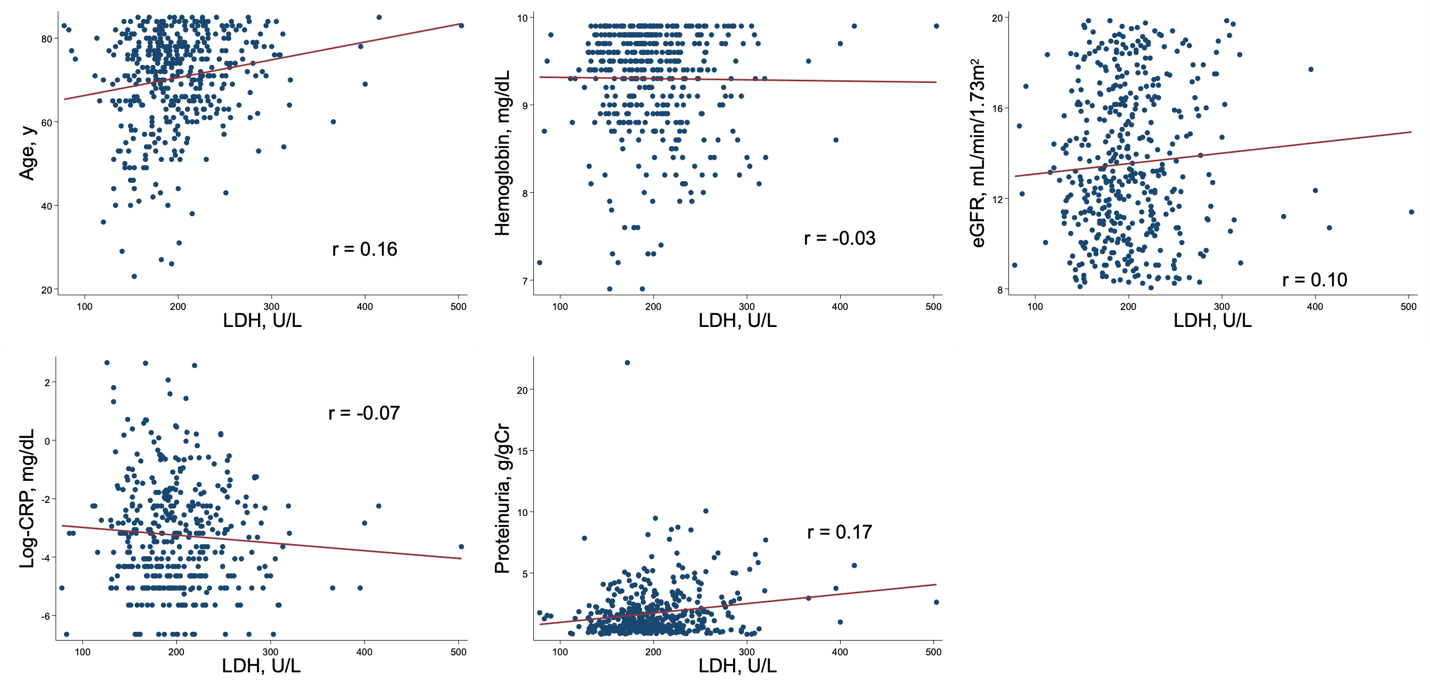


Serum LDH levels showed a weak positive correlation with age and proteinuria.

Pearson correlation was used for serum eGFR and log-CRP. Spearman correlation was used for age, serum hemoglobin, and proteinuria.

eGFR, estimated glomerular filtration rate; LDH, lactate dehydrogenase; log-CRP, log-transformed C-reactive protein. **Figure S3. Restricted cubic spline analysis to compare the association of serum LDH levels with the study outcomes**


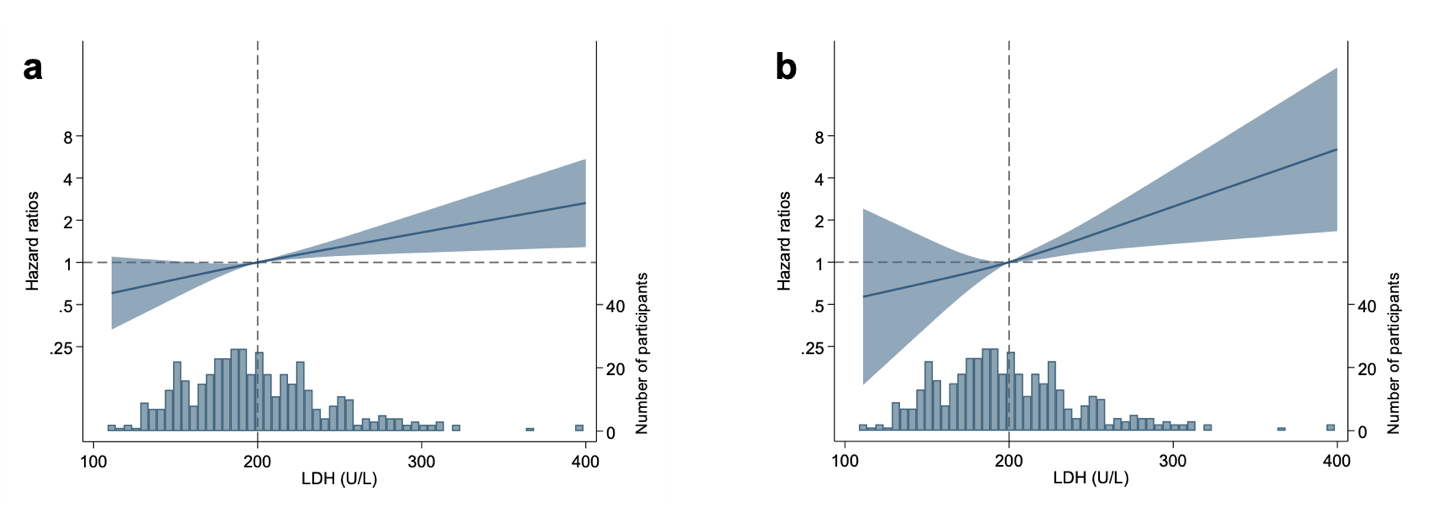


Adjusted hazard ratios of serum LDH level as continuous variables for renal composite outcomes (a) and the composite of CV events or death (b). The references was set at 200 U/L for serum LDH level. The multivariable-adjusted model was adjusted for age, sex, history of cardiovascular disease, estimated glomerular filtration rate, hemoglobin, C-reactive protein, proteinuria, and high-hemoglobin treatment group.

LDH, lactate dehydrogenase.
